# Magnetic Detection Structure for Lab-on-Chip Applications Based on the Frequency Mixing Technique

**DOI:** 10.3390/s18061747

**Published:** 2018-05-29

**Authors:** Amine Rabehi, Benjamin Garlan, Stefan Achtsnicht, Hans-Joachim Krause, Andreas Offenhäusser, Kieu Ngo, Sophie Neveu, Stephanie Graff-Dubois, Hamid Kokabi

**Affiliations:** 1 Laboratoire d’Electronique et d’Electromagnétisme, Sorbonne Université, L2E, 75252 Paris, France; amine.rabehi@sorbonne-universite.fr (A.R.); benjamin.garlan@gmail.com (B.G.); hamid.kokabi@sorbonne-universite.fr (H.K.); 2 Institute of Bioelectronics (ICS-8), Forschungszentrum Jülich, 52428 Jülich, Germany; s.achtsnicht@fz-juelich.de (S.A.); a.offenhaeusser@fz-juelich.de (A.O.); 3 Laboratoire Interfaces et Systèmes Électrochimiques, LISE, Sorbonne Université, CNRS, F 75005 Paris, France; kieu.ngo@sorbonne-universite.fr; 4 PHENIX, Sorbonne Université, CNRS, F 75005 Paris, France; sophie.neveu@sorbonne-universite.fr; 5 Faculte de Medecine, Sorbonne Université, CIMI-PARIS, UMRS CR7-Inserm U1135-CNRS ERL 8255, 75013 Paris, France; stephanie.graff-dubois@sorbonne-universite.fr

**Keywords:** Lab-on-Chip, magnetic sensing, frequency mixing, superparamagnetic nanoparticles, magnetic beads, microfluidics

## Abstract

A magnetic frequency mixing technique with a set of miniaturized planar coils was investigated for use with a completely integrated Lab-on-Chip (LoC) pathogen sensing system. The system allows the detection and quantification of superparamagnetic beads. Additionally, in terms of magnetic nanoparticle characterization ability, the system can be used for immunoassays using the beads as markers. Analytical calculations and simulations for both excitation and pick-up coils are presented; the goal was to investigate the miniaturization of simple and cost-effective planar spiral coils. Following these calculations, a Printed Circuit Board (PCB) prototype was designed, manufactured, and tested for limit of detection, linear response, and validation of theoretical concepts. Using the magnetic frequency mixing technique, a limit of detection of 15 µg/mL of 20 nm core-sized nanoparticles was achieved without any shielding.

## 1. Introduction

The detection of a biological agent or entity and the determination of its concentration have become paramount for anticipating possible health threats, whether epidemic or pandemic, environmental threats, or to combat other contextual threats, including bioterrorism, or chemical and biological weapons. Additionally, the ever-increasing number of people traveling on airplanes leads to the rapid spread of diseases worldwide and enhanced risks of pandemics. In this field, one of the main objectives is to facilitate detection with a rapid, sensitive, reliable, low-cost, and portable system for nomadic applications of pathogen detection such as Mobile Point-of-Care (MPoC). To achieve this objective, designing and generating embedded microsystems composed of innovative sensors is necessary [1,2]. Many attempts have been made at developing a LoC system. Among them, electrochemical and optical based methods dominate; however, they often suffer from drawbacks such as a nonlinear detection range, a lack of sensitivity, non-specific detection, and complexity of integration [3,4,5]. Other methods include mechanical [6,7] and magnetic detection approaches. The latter holds promise due to their miniaturization possibility [8,9,10,11]. Many attempts have been made to embed magnetic sensors such as Giant Magnetoresistance Effect (GMR) based [10], chip-based nuclear magnetic resonance (µNMR), and planar Hall Effect sensors [11,12,13,14]. µNMR systems rely on the detection of magnetic nanoparticles markers based on the proton signal and can achieve high sensitivity due to its intrinsic signal amplification properties. Inconveniences include compensating for the temperature drift and the need for a field greater than 0.1 T produced by a heavy permanent magnet that decreases the portability of the system. GMR and Hall Effect based techniques rely on the advance of microfabrication processes. Their miniaturization is thus facilitated, and they can achieve multiplexed detection through the use of an array of GMR sensors. Both techniques suffer from requiring clean room technology, nonlinearity (GMRs), and the relative difference between sample and sensor sizes that may lead to errors if the markers are unevenly distributed in the sample area, as is the case with lateral flow assays. Other drawbacks include magnetic nanoparticles residues sticking on the edges of the magnetic material and hysteresis effects.

Other magnetic biosensing techniques include dynamic techniques based on the Giant Magneto-Impedance (GMI) effect [15,16,17]. With this method, impressive detection limits have been achieved. However, using magnetic materials in the sensor element may exhibit remanence that may lead to unwanted magnetic capture of the magnetic particles.

Our envisioned LoC system is based on the development of a simple and cost effective system using magnetic detection, taking advantage of the nonlinearity of superparamagnetic nanoparticles (SPN). Biological analysis with high sensitivity magnetic measurements is a new type of immunochemical diagnosis using SPN as markers instead of conventional enzymes, radioisotopes, or fluorescent compounds [18,19]. This novel analysis method can be miniaturized and involves the covalent ligation of biomarker-specific monoclonal antibodies to SPN. The presence of SPN is then detected by the frequency mixing method that involves the use of two distinct excitation frequencies and the reading and quantification of mixed frequency signals indicative of magnetic non-linearity of the superparamagnetic nanoparticles [20,21,22]. A similar inductive approach using the frequency in the Megahertz regime was introduced [23]. This technique enabled the creation of a rapid, portable, and cheaper detection method compared with the other major detection techniques. With all its components, the sensor should be fabricated only from diamagnetic or paramagnetic materials that have linear magnetic characteristics in the relevant magnetic field range of a few Millitesla. By entirely avoiding ferromagnetic materials, frequency mixing components can be generated by SPN with their nonlinear magnetization curve. Thus, background signals can be efficiently avoided and a high sensitivity and specificity to SPN can be achieved.

In this work, the detection system was optimized using the planar pick-up and excitation coils design for sensitive detection and efficient magnetization. Furthermore, practical aspects, such as heating and parasitic effects that can cause substantial damage to the biological samples, were considered. Heating can cause both a decrease and fluctuation in the detected signal since the magnetization of the SPN is inversely proportional to the temperature [24]. Consequently, compromises should be considered for this miniaturized system.

Here, we propose the use of simple planar coils for the precise detection of magnetic nanoparticles in a miniaturized system. We present a magnetic sensing miniaturization scheme including analytical calculations and simulations. We then validate the analytical calculations with a Printed Circuit Board (PCB) microfluidic prototype. This prototype is characterized with respect to its electromagnetic properties and its sensitivity to a sample of SPN using a personalized test bench. At last, we discuss the results, the envisioned applications of this prototype, and its further miniaturization using the proposed methodology.

## 2. Design and Optimization of the Structure

**Figure 1:**
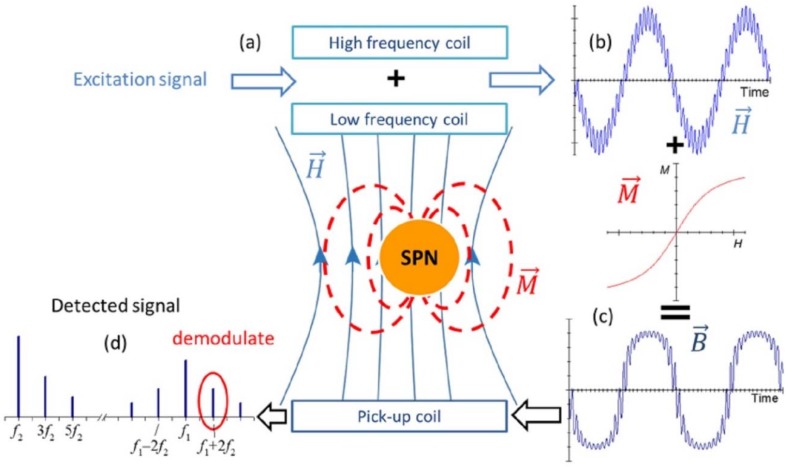
(**a**) Principle of detection of the proposed structure; (**b**) magnetic excitation field; (**c**) resulting magnetic flux density; and (**d**) Fourier transform of the detected signal. In red, the selected mixing term for the detection of SPN ($f_{1}+2f_{2}$) is marked.

The mixing frequency technique involved using two sinusoidal signals of frequencies *f*_1_ and *f*_2_ generated by high frequency (HF), and low frequency (LF) coils, respectively. These coils generated an excitation magnetic field that allowed the magnetization of SPN enclosed in a sample microfluidic reservoir (Figure 1a,b). Due to the nonlinearity and saturation properties of SPN, the resulting magnetic flux density included mixing components, $n_{1}f_{1}\pm n_{2}f_{2}$. These harmonics allow specific distinction of SPN (Figure 1c). Consequently, after detecting the magnetic flux density using the inductive pick-up coil, the signal was demodulated at the desired mixing term (Figure 1d).

The aim of this work was to design and develop a miniaturized device based on the proposed method of detection. To achieve this goal, all three integrated planar coils should be optimized with respect to their inner parameters (frequency, current, noise, sensitivity, etc.) as well as the parameters of the structure as a whole (temperature, mutual inductance, balancing efficiency, etc.). This section explains the methodology and presents the important factors to consider for magnetic sensing in general and LoC biosensors in particular.

### 2.1. Pick-Up Coil Optimization

The proposed detection method relies on the unique nonlinear response of the SPN. Consequently, the sensor must not contain any ferromagnetic components. For this reason, we chose to operate with an air cored coil instead of iron cored coils. Air cored coils are used in many applications, including industrial, geophysical, and biomedical applications [25]. Advances in microelectromechanical systems (MEMS) design and microfabrication allow the design of miniaturized micro-coils. However, finding the optimal dimensions for a miniaturized coil remains challenging; the fabrication method, the practical distance from coil to sample, and the area of detection are all different factors that result in upper and lower dimension limits for the desired coil. Thus, our calculations were restricted to planar spiral coils. The two main aspects of a pick-up coil are sensitivity and noise, from which the minimum detectable magnetic moment and the magnetic moment sensitivity can be determined.

The sensitivity of a coil with respect to detecting nanoparticles is defined here as the ratio of the induced voltage to the magnetic moment that caused it:

$$S_{sensor}=\frac{U_{ind}\left(Z\right)}{m_{z}}$$

where $m_{z}=m_{0}\cdot \sin\left(2\pi ft\right)$ is the total magnetic moment of all SPN. This approximation holds because each SPN is a single domain particle and all the nanoparticles exhibit the same magnetization response in both amplitude and direction when subjected to the same magnetic excitation field. 

The induced voltage in a single wire loop of radius $R_{1}$, caused by an oscillating magnetic moment $m_{z}$ at a distance Z from the loop can be expressed as

(1) $$U_{\left(z,t\right)}=-\frac{d\Phi }{dt}=\frac{1}{2R_{1}}\frac{\mu_{0}}{{\left(r^{2}+z^{2}\right)}^{\frac{3}{2}}}\cdot \frac{dm_{z}}{dt},$$

where $r=R/R_{1}$ and $z=Z/R_{1}$ are dimensionless parameters relative to the inner radius $R_{1}$ of the coil. Integration over the width of the spiral coil results in:

(2) $$U_{0}=\frac{N}{w}\int_{1}^{1+w}U\left(z,t\right)dw=m_{z}\mu_{0}2\pi f\frac{N}{2R_{1}}\cdot G\left(z,w\right),$$

where

(3) $$G\left(z,w\right)=\frac{1}{w}\left[\frac{1}{\sqrt{1+z^{2}}}-\frac{1+w}{\sqrt{{\left(1+w\right)}^{2}+z^{2}}}+\ln\left(\frac{1+w+\sqrt{{\left(1+w\right)}^{2}+z^{2}}}{1+\sqrt{1+z^{2}}}\right)\right]$$

is the geometrical function representing the effect of the pick-up coil dimensions. From this, we can deduce the total sensitivity for $N_{l}$ layers:

(4) $$S_{sensor}=\mu_{0}2\pi f\frac{N}{2R_{1}}\cdot \sum_{i=1}^{N_{l}}G\left(z_{i},w\right).$$

where $z_{i}$ denotes the distance of each spiral layer to the magnetic moment (sample location).

With regard to noise, the aim was to approach the minimum detectable magnetic moment that is defined by the moment that can be detected when noise and signal become equal (SNR=1). Since the first preamplifier stage was considered, this measurable value was used to compare the efficiency of various sensors with respect to detecting magnetic beads. 

Generally, a detection system includes a preamplification stage, a damping resistance ($R_{p}$), and a parallel capacitance ($C_{p}$). A coil can be represented electrically by its lumped parameters inductance $L_{s}$, AC resistance $R_{AC}$, and parasitic capacitance $C_{S}$. As stated in Choi et al. [2], the total noise voltage can be estimated as:

(5) $$U_{N}=\sqrt{\frac{U_{a}^{2}}{{\left|TF\left(f\right)\right|}^{2}}+\left(\frac{4k_{B}T}{R_{P}}+I_{a}^{2}\right)\cdot {\left|R_{S}+i2\pi fL_{S}\right|}^{2}+4k_{B}TR_{S}}.$$

where $U_{a}$ and $I_{a}$ are the voltage and current noise of the preamplifier, respectively, and TF(f) is the transfer function of the detection circuit. All the electrical parameters should be optimized so that the Johnson noise dominates and only the sensor noise remains. Consequently, we optimized the sensor coil to have maximum sensitivity with the least resistance and lowest inductance values. A high inductance value will result in a high current noise contribution.

The corresponding magnetic flux noise density $B_{N}$ can be deduced by multiplying the total noise voltage by the overall coil sensitivity,

(6) $$B_{N}≅\frac{2}{\pi^{2}N_{l}N\cdot D^{2}f}U_{N},$$

where $N_{l}$ denotes the number of layers and D the average diameter of the coil.

Two values of interest can be deduced: the signal to noise ratio (SNR) and the minimum detectable magnetic moment. 

In the case where the choice of readout electronics is correctly performed, we suppose that the *SNR* is the ratio of the detected signal versus the thermal noise, $U_{N}=\sqrt{4k_{B}T∆fR_{S}}$, with $R_{S}=R_{skin}+R_{DC}+R_{proximity}≅R_{DC}$. Since the skin depth for our maximum frequency is around 200 µm, the skin and proximity effects are negligible. This assumption will be validated in the results section. Thus,

(7) $$U_{N}=4\sqrt{\frac{\rho \pi k_{B}T}{H_{wire}}}\cdot \frac{\sqrt{1+\frac{w}{2}}}{\sqrt{w}},$$

where $\rho ,k_{B},\;T,\;H_{wire}$ denote the resistivity, Boltzmann constant, absolute temperature, and height of the wire section, respectively.

The *SNR* is then:

(8) $$SNR=\frac{U_{ind}}{\sqrt{2}U_{N}}=m_{z}\frac{{\text{µ}}_{0}\sqrt{\pi }}{4\sqrt{\rho k_{B}T}}f\cdot \left[\frac{\sqrt{H_{wire}}}{R_{1}}\cdot \frac{\sum_{i=1}^{N_{l}}G\left(z_{i},w\right)}{N_{l}\sqrt{w}\sqrt{1+\frac{w}{2}}}\right].$$

Whereas *SNR* is directly related to the geometry of the coil, the frequency, and the physical parameters, the minimum detectable moment provides a physical limit that can be linked to the amount and concentration of a given number of nanoparticles. The moment that corresponds to the *SNR* in a given bandwidth Δf is obtained as:

(9) $$\frac{m_{z}}{\sqrt{∆f}}=\frac{4\sqrt{\rho k_{B}T}}{{\text{µ}}_{0}\sqrt{\pi }}\cdot \frac{1}{f}\left[\frac{R_{1}}{\sqrt{H_{wire}}}\cdot \frac{N_{l}\sqrt{w}\sqrt{1+\frac{w}{2}}}{\sum_{i=1}^{N_{l}}G\left(z_{i},w\right)}\right].$$

Consequently, we can detect smaller magnetic moments if we use higher frequencies and a smaller inner radius.

**Figure 2:**
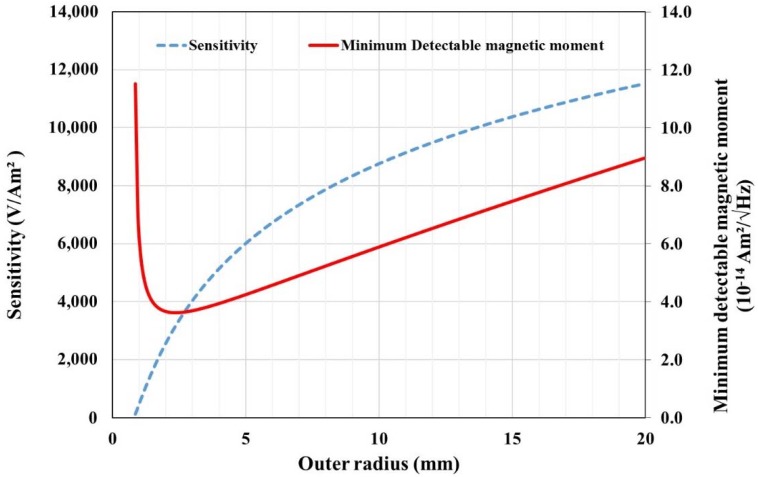
Pick-up coil optimization with sensitivity and minimum detectable moment versus coil outer radius. Inner radius was fixed at 0.8 mm.

Finally, we can optimize the pick-up coil’s dimensions by finding a compromise between sensitivity and the minimum detectable moment. Figure 2 shows the best sensing characteristics that we obtained by applying PCB restrictions to the copper section, interlayer distances, and minimal practical inner radius.

For an outer radius $R_{out}>$ 2 mm, a region where the added noise, due to increasing number of turns, becomes predominant with regard to the improvement in sensitivity.

### 2.2. Excitation Coils Optimization

Pick-up coil optimization should be performed in conjunction with that of the excitation coils. Magnetic excitation of SPN is receiving considerable interest. In fact, the limit of detection in terms of the number of SPN can be smaller than expected if the SPN are not properly magnetized.

Since the magnetic frequency mixing technique relies on the detection of the nonlinear response of SPN, the magnetic field amplitude should therefore be adapted. As explained in Choi et al. [2], the low frequency field ($f_{2}$) is used as a drive field and the high frequency field ($f_{1}$) is used as an excitation field. For relatively small magnetic fields, the amplitude of the mixed frequency term is proportional to the high frequency amplitude and to the square of the low frequency amplitude. Because of this, emphasis on the minimum required amplitude is directed to low frequencies, whereas for high frequencies, the frequency of use contributes more to the optimization of the system. For example, for 20 nm nanoparticles, we used 2 mT as the low frequency drive field amplitude and around 1 mT as the high frequency excitation field amplitude.

Using the generated magnetic field from single turn circular coils, we summed the effect of the different radii to determine the magnetic field density generated on the symmetry axis of the spiral coil:

(10) $$B_{z}\left(z_{i}\right)=\frac{\mu_{0}\cdot N\cdot I_{exc}}{2\cdot R_{1}}\cdot \sum_{i=1}^{i=N_{l}}G\left(z_{i},h\right)$$

where $I_{exc}$ is the excitation current. The formula was then used to find the minimum excitation current. This formula was validated with measurement tests (Gaussmeter GM08, Hirst Magnetics, Cornwall, UK) with an error less than 5%. The distribution of the magnetic field should be rather homogeneous along the radial dimension of the reservoir to achieve a homogeneous sensitivity distribution. This was confirmed by electrical simulations (COMSOL Multiphysics) of the magnetic field generated by a spiral coil. The homogeneity of the field depends on the inside and outside radius and on the distance of the reservoir from the center of the coil. To properly magnetize the SPN, we fixed the appropriate distance *Z* of the reservoir and we deduced the maximum area that it could occupy.

Because the structure was intended for immunoassays with biological samples, the electrical simulation was coupled with a heat transfer simulation also performed with COMSOL Multiphysics. The coupled physics facilitated the inclusion of both electromagnetic and heat aspects. We therefore predicted the acceptable maximum measurement time, according to the maximum allowed temperature in the reservoir for preserving biological sample’s viability (<40 °C).

Increasing the number of turns helped reduce the minimum required current; however, the added value of the external turns became negative after a certain threshold, where the number of turns increased the resistance and inductance in a linear and quadratic manner, respectively. This led to a decrease in the minimum current due to their limited contribution to the generated magnetic field.

Furthermore, the sensitivity might be deduced to be greater when the frequency is greater. In fact, the maximum excitation frequency that we could use was limited by two factors: the relaxation time of the SPN and the complexity of generating relatively high currents for the resulting high frequency impedance.

## 3. Materials and Methods

### 3.1. Magnetic Detection Device

**Figure 3:**
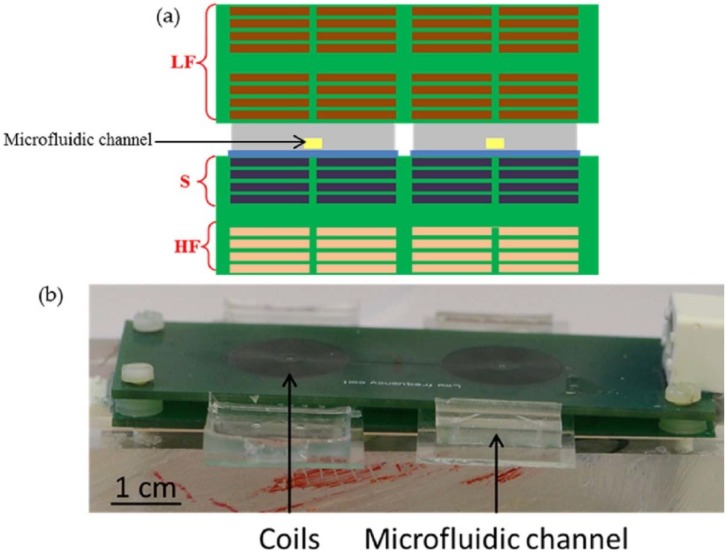
(**a**) Schematic description of the layers (front view): the two green blocks represent the two Printed Circuit Boards (PCBs) containing the coils (LF: Low Frequency, S: Sensor, and HF: High Frequency), the grey part represents the PDMS layers that contain the microfluidic channel (the yellow part), which are bonded to a glass slide (in blue); (**b**) Picture of the PCB/ microfluidic prototype detection structure.

To validate the above-mentioned calculations, a PCB/microfluidic prototype was developed. The structure was composed of 4 copper coils: three coils emit the electromagnetic field, two for low frequency, and the other for high frequency, the fourth one was the detection coil. These coils were contained in two PCB structures (100 × 40 × 1.55 mm^3^) surrounding above and under the serpentine-like microfluidic channel (12 × 12 mm^2^) that can contain 14 µL of magnetic nanoparticle solution. Each coil was composed of four layers; the tracks were 100 µm wide with an inter-distance of 100 µm. Each layer of track had a thickness of 35 µm. The emitting coils had a radius of 13 mm (60 turns/layer) and the detection coil had a radius of 10 mm (46 turns/layer). Because both the excitation and pick-up coils were made of the same PCB, we had to balance the above-mentioned criteria for proper magnetization and detection. For the validation process, the distance between the PCBs was 4 mm and the distance between the detection coil (lower PCB) and the microfluidic chamber was 1 mm. Two sets of PCB/microfluidic were used: one for the measurement and one for reference. They were linked to subtract the signal detected with the sample and the signal detected with the reference to obtain a precise measurement of the difference (Figure 3). The PCB structure was custom-made by a commercial industrial vendor.

### 3.2. Microfluidic Channel

**Figure 4:**
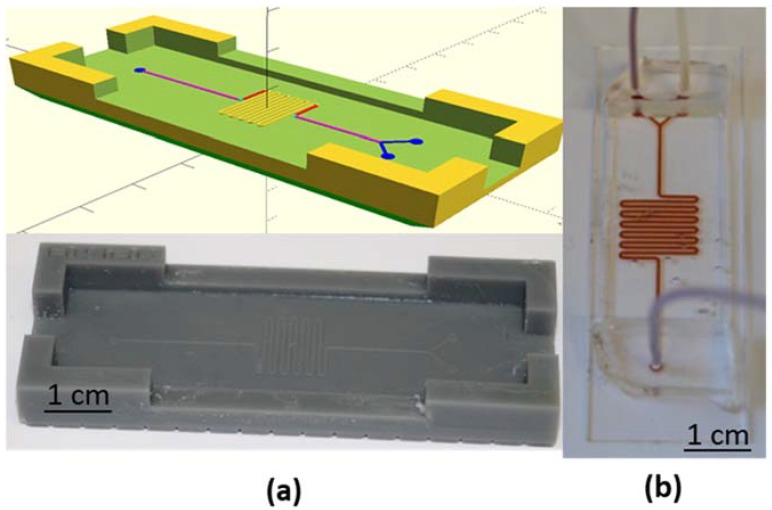
(**a**) Three-dimensional (3D) printed master mold and (**b**) microfluidic channel, filled with a highly concentrated superparamagnetic nanoparticles (SPN) solution for improved visibility.

The microfluidic channel was made using a polydimethylsiloxane (PDMS) molding technique. A serpentine 12 × 12 mm^2^ reservoir was created with a 500-µm wide channel that was 200 µm in height. A three-dimensional (3D) printer was used to create the master mold (Figure 4a). This technique is faster, simpler, and cheaper than the SU8 master mold used in classical photolithography for these dimensions. To create a channel, the liquid monomer and the curing agent (ratio 10:1) were poured on the master mold and placed in the oven at 80 °C for one hour. Then, the cured polymer was peeled off from the master mold and exposed with a glass slide under plasma O_2_ for one minute. The plasma created hydroxyl groups (–OH) on both the PDMS and the glass slide that form covalent bonds when they are pressed together (Figure 4b). After bonding, the device was kept in the oven at 80 °C for at least three hours before use. After bonding, the device was kept in the oven at 80 °C for at least three hours before use.

The serpentine shape was chosen because of its improved mechanical stability in comparison to an ovoid reservoir. In addition, this structure yields an increased surface area, which can be used for surface functionalization.

As shown in Figure 4, the thickness of the PDMS was reduced to 1.5 mm (plus the 1 mm thickness of the glass slide) in the middle part of the channel to bring the coils and the magnetic nanoparticles in the channel as close together as possible. At the extremities of the channel, a larger PDMS thickness of 5.5 mm (plus 1 mm glass) was needed to connect the inlet and outlet tubing.

### 3.3. Test Bench

**Figure 5:**
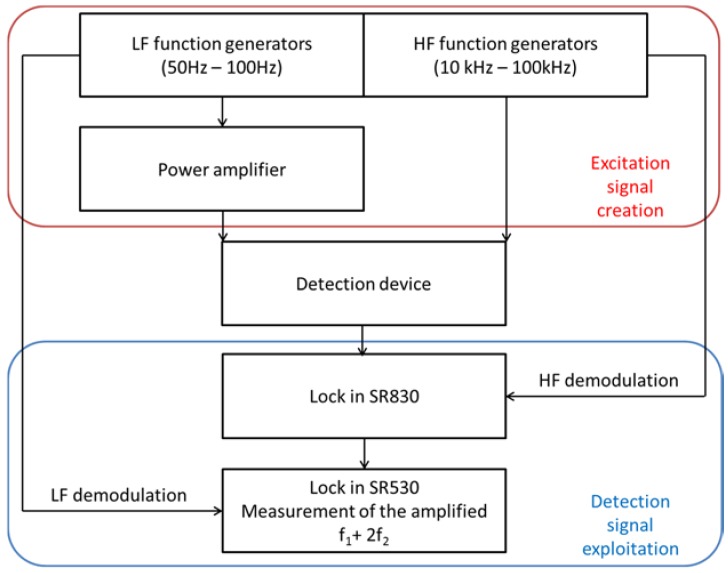
Outline chart of the measurement test bench.

The chart of the measurement test bench is shown in Figure 5. Two sinusoidal signals were generated by function generators to create the low and high frequency magnetic fields. The low frequency signal was amplified to reach the desired magnetic force. The detection coils in the device converted the measured magnetic field into an electric signal that was sent to the first lock-in, which demodulated and amplified the signal by removing the high-frequency component of the response. The second lock-in removed the low frequency twice so that the harmonic *f*_1_ + 2*f*_2_ was isolated and measured.

## 4. Results and Discussion

### 4.1. Characterization of the Structure

In the aforementioned calculations described in Section 2, we estimated the parameters related to sensitivity, noise, and the excitation magnetic field. The calculations were partially based on an estimation of the electrical lumped parameters of the PCB coils, such as the resistance and inductance. Concerning the inductance, we observed a 6% maximum error between the calculated and measured values, which is an acceptable value for our optimization scheme. For example, the sensor calculated and measured inductances were 490 and 520 μH, respectively. This leads to an acceptable error rate of 5.7%.

**Figure 6:**
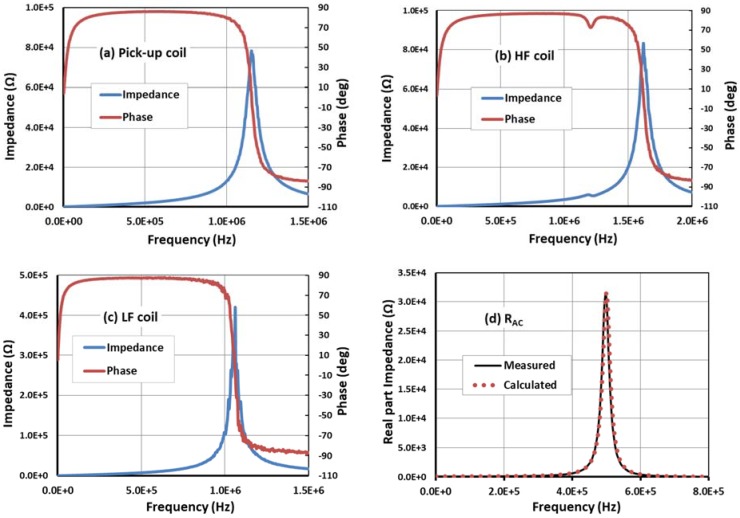
Effect of the frequency on the impedance of the (**a**) pick-up; (**b**) HF; and (**c**) LF coils; and (**d**) effect of the operating frequency on the AC resistance.

To assess the effect of the parasitic capacitance on the self-resonance of the coils and the effects of AC on the value of the resistance, the fabricated PCB coils were characterized using an impedance analyzer (4194, Hewlett Packard, Palo Alto, CA, USA). The impedance and the phase changes of the different coils were characterized over a spectrum from a few kHz and 2 MHz (Figure 6).

The electrical behavior of the excitation and pick-up coils are shown in Figure 6a–c. We observed that the resonance frequency was above 1 MHz for all coils. For the LF coil, this frequency was a little bit lower since it was composed of eight layers. Consequently, the LF coil had a higher inductance value, but this was not an issue since it operated at low frequencies, which was considered during the optimization process. The HF and pick-up coils had higher resonance frequencies and less inductance, which permitted a stronger magnetic field at higher frequencies. The mutual effects were insignificant but should be considered for smaller coil dimensions and distances between coils. For example, a mutual effect was observed on the HF coil due to its proximity to the sensor coil (fabricated on the same PCB) that resulted in a secondary peak. 

Moreover, we assessed the effect of the frequency on the AC resistance by plotting the evolution of the measured and calculated real part of the impedance, assuming that *R* was constant throughout the target spectrum ($R_{coil}=R_{DC}$). The real part was deduced from the *RLC* series equivalent electrical model of a coil,

(11) $$Z_{Sensor}\left(real\right)=\frac{R}{{\left(1-\omega^{2}LC\right)}^{2}+{\left(\omega RC\right)}^{2}},$$

where ω=2πf represents the angular frequency. As can be seen in Figure 6d, the measured and calculated real part impedance were very similar, which means that the AC effects do not have any important impact on the behavior of the coil at frequencies below 1 MHz. All electrical characteristics of the structure are summarized in Table 1.

Finally, for the parasitic capacitance of the coils, the calculations had to consider the dielectric properties of the materials used in the PCB coils, the interlayer spacing, trace spacing, and other dimensional parameters. However, we could reduce this parasitic effect by reducing the capacitance magnitude due to interlayer distance by ensuring the trace lines of the two adjacent levels were not parallel to one another. The latter consideration allowed us to shift the sensor resonance frequency from 0.54 to 1.16 MHz.

The entire structure was characterized using the developed test bench. One of the important factors was the gap distance between the upper and lower PCBs that translated into a greater distance between the coils and the sample. Naturally, the bigger this distance, the worse the sensitivity relative to the SPN concentration. For example, in the proposed structure, the sensitivity ratio was about 1.5 between distances of 2.4 and 3.2 mm. This is explained by the weaker low frequency driving field that is quadratically related to the mixing term measured signal, $A\left(f_{1}+2f_{2}\right)\propto A{\left(f_{2}\right)}^{2}$ [2].

As balancing is critical for noise reduction, we approximated the balancing ratio of the two realized prototypes. For this, we tested the PCBs in both balanced and unbalanced configurations. The observed balancing ratio was about 1000 for the proposed prototype. This good balancing ratio allowed the system to be more sensitive to the mixing term signals. The balancing ratio could be further improved by more mechanical balancing of the horizontal angle of the PCB (upper) with respect to the other PCB (lower). 

Concerning the effect of the high frequency change in a fixed input voltage situation, we observed that the increase in frequency did not significantly contribute to the sensitivity. This can be explained by the fact that the amplification stage delivers a constant voltage output in the operation range. When the frequency $f_{1}$ increases, the impedance of the sensor increases, which reduces the excitation current and thus the magnetic excitation field $A\left(f_{1}\right)$, yielding a decrease in the response of the SPN, $A\left(f_{1}+2f_{2}\right)\propto f_{1}$ and $A\left(f_{1}+2f_{2}\right)\propto A\left(f_{1}\right)$.

### 4.2. Sensing Performance of the Device with SPN

The sensitivity response of the structure was then tested with respect to the concentration of the SPN For these experiments, the low frequency was 80 Hz with a coil voltage of 14 V and the high frequency was 40 kHz with a voltage of 15 V. The response signal of the detection coil was amplified by a factor of 500 using a standard lock-in amplifier (Stanford Research SR830, SRS, Sunnyvale, CA, USA). Different concentrations of iron oxide nanoparticle (Fe_2_O_3_) suspensions were used to validate the detection technique and determine the limit of detection (LOD) of the structure. The core of the SPN synthesized by the PHENIX laboratory [26] had a mean diameter of 20 nm. Their flower-like structure offers stability at such large diameters, resulting in a higher magnetic moment response (Supplementary Materials).

**Figure 7:**
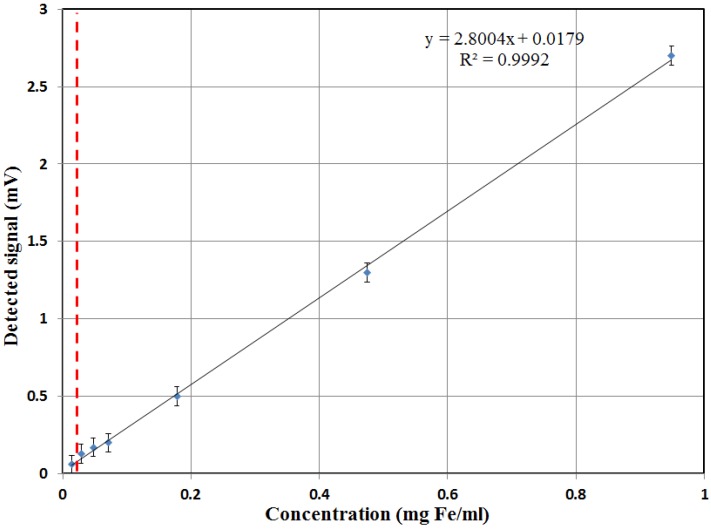
Magnetic response as a function of the mass concentration of 20 nm iron oxide (Fe_2_O_3_) nanoparticles. The red dotted line indicates the calculated limit of detection.

The magnetic response as a function of the mass concentration of iron oxide nanoparticles is shown in Figure 7. The amplitude of the detected signal is linear with the concentration of the nanoparticles for a linear range of three orders of magnitude (*R*^2^ = 0.999). Furthermore, the tests were repeated at one-day intervals and proved to be very reproducible. With an accepted error of less than 15%, the limit of detection was about 15 µg/mL.

**Figure 8:**
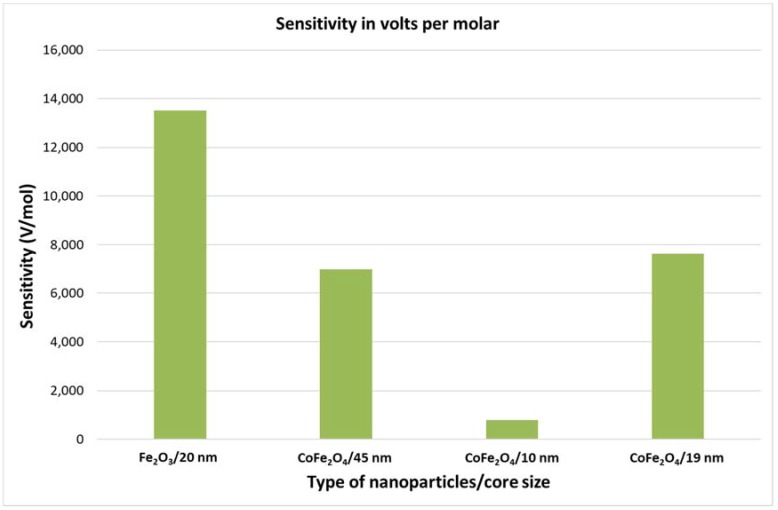
Sensitivity of the magnetic detection per molar quantity of the different nanoparticles. Details on the type of particles are provided in the Supplementary Materials.

Other types of nanoparticles composed of different metallic components and with different core sizes were also tested. Detailed information on the magnetic particles is provided in the Supplementary Materials, including a table summarizing the particle data, transmission electron microscopy (TEM) images, measured and fitted size distribution curves, and M(H) loops. Figure 8 shows the sensitivity of the device as a function of the types of nanoparticles. We can observe that the sensitivity of the device is worst for the smallest nanoparticles which can be due to the poor surface magnetization effects (spin-canting effects). On the other hand, the size does not seem to affect the magnetization since the sensitivity is almost the same for CoFe_2_O_4_ nanoparticles of 45 nm and 19 nm. This might be due to the difference in synthesis procedure, since the 45 nm and the 19 nm CoFe_2_O_4_ nanoparticles were synthetized through polyol and hydrothermal processes, respectively. Furthermore, the composition of nanoparticles is more important because the sensitivity for 20 nm Fe_2_O_3_ nanoparticles was higher than that for 19 nm CoFe_2_O_4_ nanoparticles, which can be explained by a better nonlinearity of Fe_2_O_3_ nanoparticles. Consequently, the device has the best sensitivity with the iron oxide nanoparticles of 20 nm were used for the experiments.

## 5. Conclusions

Our preliminary results are very promising as they confirm the ability to magnetically detect SPN within the developed prototype device. Experiments to validate the magnetic detection of biological entities with the PCB structure are in progress.

Additional noise sources, both internal and external, must be optimized since they increase the difference between the theoretical and experimental LOD. The internal noise sources include electrical noise from excitation circuit that results in crosstalk, white magnetic field noise, and a small temperature gradient that slightly changes the magnetization. The diffusion heterogeneity of ferrofluids was also a contributing factor to the error bars shown in the performance curve in Figure 7. External interfering signals can be in the form of magnetic field disturbances from adjacent power sources and electronic instruments.

Future improvements will focus on improving the LOD and minimizing the sample volume by using clean room technology. This should allow the positioning of the detection coils closer to the sample area with higher precision, thus allowing a better LOD for smaller sample volumes. The proposed method of detection can be coupled with magnetic actuation techniques, provided that no ferromagnetic material is used in the vicinity of the detection area.

Different realized PCB prototypes allowed experimental validation of our methodology for optimization and consequent further miniaturization. The prototypes helped to validate and improve the electromagnetic simulations and analytical calculations tools, especially in the case of spiral coils. Also, the simplicity offered by the PCB coils allows the rapid integration of both coils and electronic accompanying circuitry, reaching the goal of a low-cost embedded magnetic detection portable system, only using off-the-shelf technologies without any complex fabrication procedures. 

This cost-effective and downsized device is already able to detect magnetic nanoparticles in 14 µL samples of any liquid. The next step is to adapt this prototype so that rapid LoC magnetic immunoassays can be performed for environmental or healthcare purposes.

## 6. Patents

This work served as a basis for a European patent proposal under the number: 17306381.9. Date of the deposition: 12 October 2017.

## Figures and Tables

**Table 1:** Electrical parameters values for two different prototypes of realized Printed Circuit Board (PCB) multilayer coils. The capacitance was deduced from the self-resonance.

Coils	Resistance (Total) (Ω)	Inductance (mH)	Capacitance (Calculated) (pF)	$f_{Resonance}$ (MHz)
LF ($f_{2}$)	160	1.8	12	1.06
HF ($f_{1}$)	68	0.4	33	1.62
Sensor	80	0.52	39	1.16

## Supplementary Materials

The following are available online at http://www.mdpi.com/1424-8220/18/6/1747/s1.
