# Supplementary material for: Magnetic Detection Structure for Lab-on-Chip Applications Based on the Frequency Mixing Technique
Source: Sensors (Basel). 2018 May 29;18(6):1747. doi: 10.3390/s18061747 (PMC6021809; doi:10.3390/s18061747)
Supplement: Supplementary file 1 [file sensors-18-01747-s001.pdf]

Supplement

Magnetic Detection Structure for Lab-on-Chip applications based on the frequency mixing technique

Typically, the core sizes of magnetic nanoparticles exhibit a log-normal size distribution [1]. The lognormal distribution function is given by

$$f(d)=\frac{1}{\sqrt{2\pi}\sigma\, d}\exp\left[-\frac{\ln^2\left(d/d_0\right)}{2\sigma^2}\right] \qquad \text{(S1)}$$

where d_0 denotes a size parameter (different from the average size) and σ a dimensionless parameter characterizing the width of the distribution.

γ-Fe_2O_3/20 nm

Fig. S1 shows a transmission electron microscopy (TEM) image of the particles composed of Maghemit (γ-Fe_2O_3). The particle core sizes were determined by image processing. Their size distribution is depicted in Fig. S2. A lognormal curve, see Eq. (S1), was fitted to the data. It is shown as a red curve in Fig. S2. The resultant parameters of the fit were d_0 = 18.1 nm and σ = 0.43. The measured magnetization of the particles as a function of the applied magnetic field is shown in Fig. S3. No hysteresis was observed when ramping the field up and down.

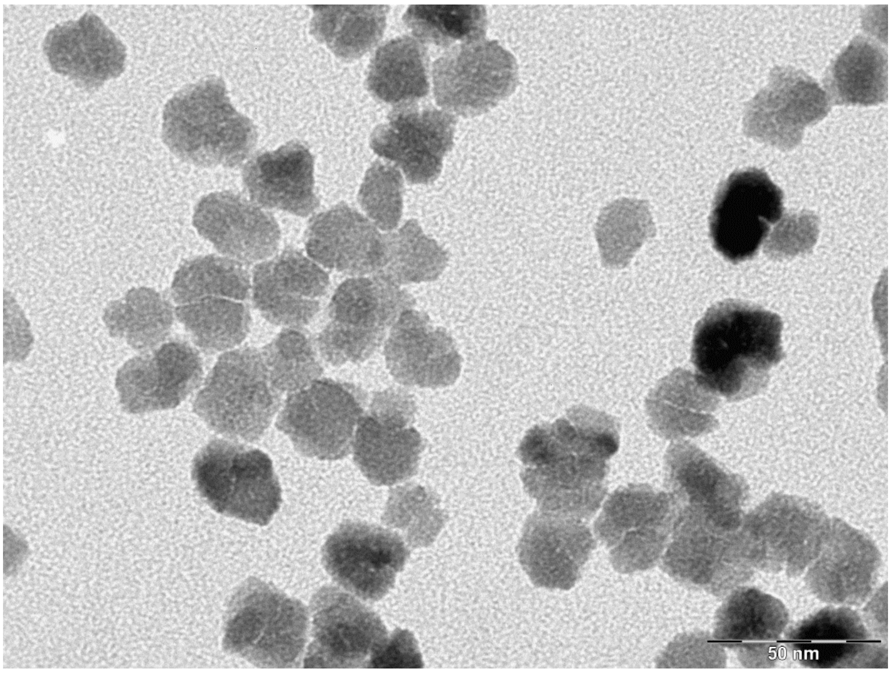

Fig. S1. Transmission electron microscopy (TEM) image of γ-Fe_2O_3/20 nm particles.

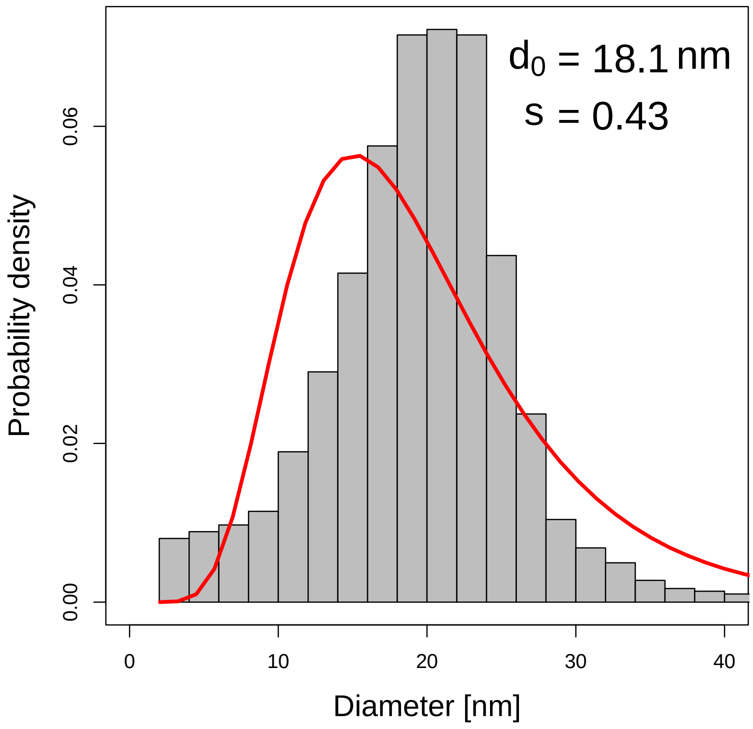

Fig. S2. Size distribution of γ-Fe_2O_3/20 nm particles. The red curve is a fit of a lognormal curve to the data.

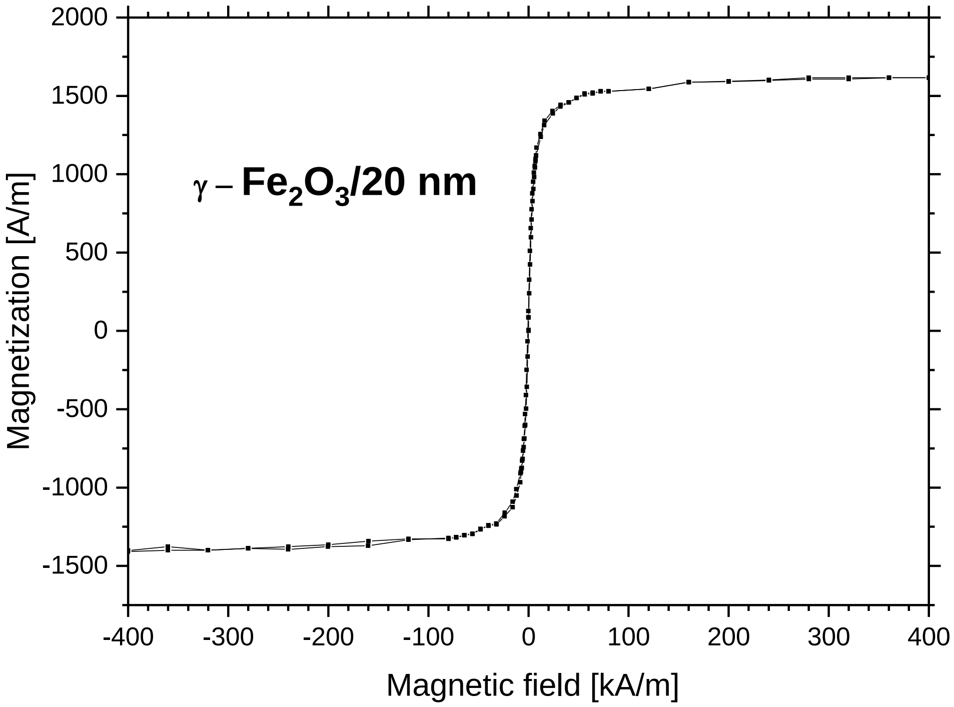

Fig. S3. Measured magnetization curve of γ-Fe_2O_3/20 nm particles with a concentration of 21.2 mg/ml.

Flower-like $CoFe_2O_4$/45 nm particles synthesized by the polyol process

Nanoflower-shaped $CoFe_2O_4$ particles of approximately 45 nm size were synthesized using the polyol process [2]. Fig. S4 shows a transmission electron microscopy (TEM) image of the particles composed of Cobalt-Ferrite ($CoFe_2O_4$). A size histogram of the individual particles cannot easily be determined because the nanoflower-shaped nanoparticles are aggregating. Details on the synthetization and characterization of these particles, including magnetization curves, can be found in Ref. [2]. Similar approaches have been also published elsewhere [3].

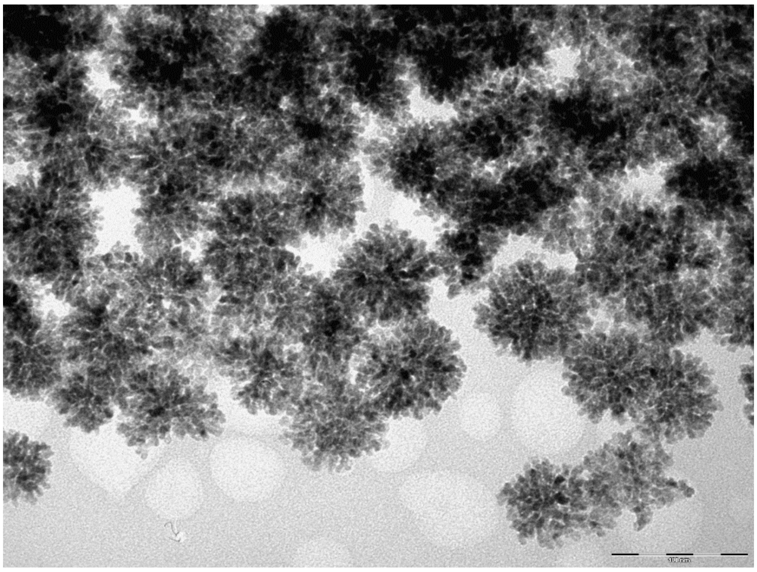

Fig. S4. Transmission electron microscopy (TEM) image of $CoFe_2O_4$/45 nm particles.

$CoFe_2O_4$/10 nm particles synthesized by the SWC process

10 nm sized $CoFe_2O_4$ particles were synthesized using the SWC chloride-based process with a Co / Fe ratio of 0.64. Fig. S5 shows a transmission electron microscopy (TEM) image of the particles composed of Cobalt-Ferrite ($CoFe_2O_4$).

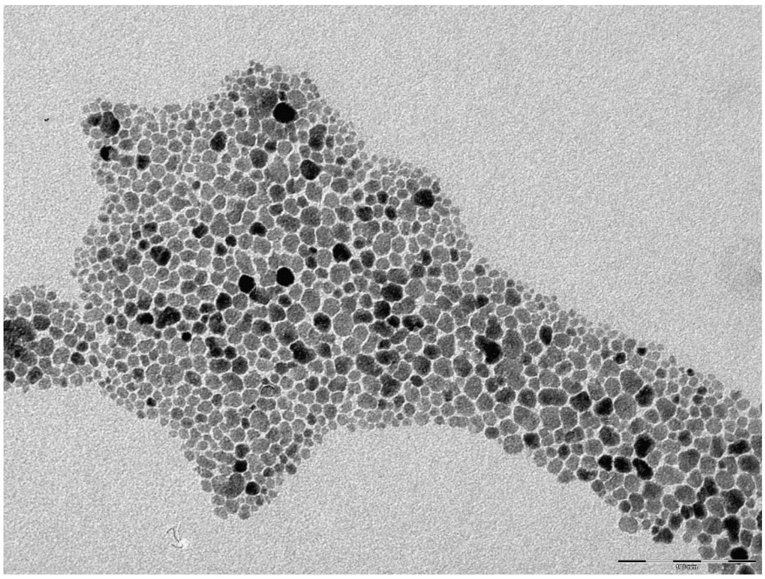

Fig. S5. Transmission electron microscopy (TEM) image of $CoFe_2O_4$/10 nm particles.

The particle core sizes were determined by image processing. Their size distribution is depicted in Fig. S6. A lognormal curve, see Eq. (S1), was fitted to the data. It is shown as a red curve in Fig. S6. The resultant parameters of the fit were d_0 = 10.2 nm and σ = 0.39. The measured magnetization of the particles as a function of the applied magnetic field is shown in Fig. S7. A slight hysteresis was observed when ramping the field up and down.

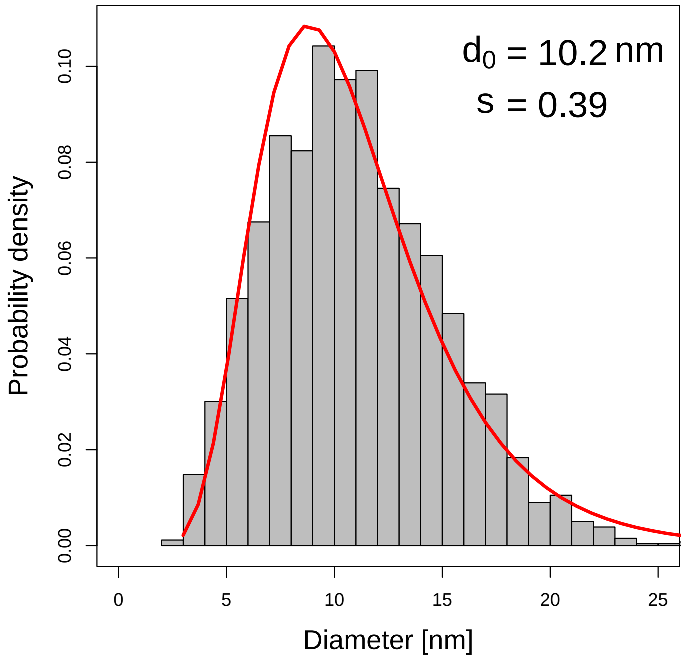

Fig. S6. Size distribution of $CoFe_2O_4$/10 nm particles. The red curve is a fit of a lognormal curve to the data.

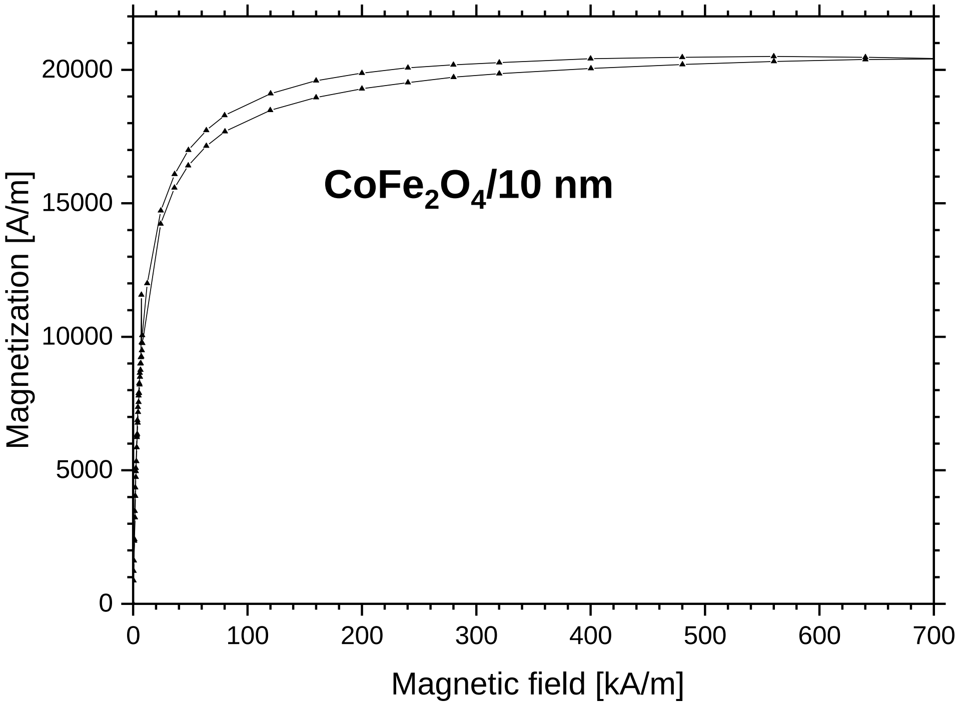

Fig. S7. Measured magnetization curve of $CoFe_2O_4$/10 nm particles with a concentration of 263 mg/ml.

$CoFe_2O_4$/19 nm particles synthesized by the hydrothermal process

19 nm sized $CoFe_2O_4$ particles were synthesized using the hydrothermal process. Fig. S8 shows a transmission electron microscopy (TEM) image of the particles composed of Cobalt-Ferrite ($CoFe_2O_4$). The particle core sizes were determined by image processing. Their size distribution is depicted in Fig. S9. A lognormal curve, see Eq. (S1), was fitted to the data. It is shown as a red curve in Fig. S9. The resultant parameters of the fit were d_0 = 18.8 nm and σ = 0.4.

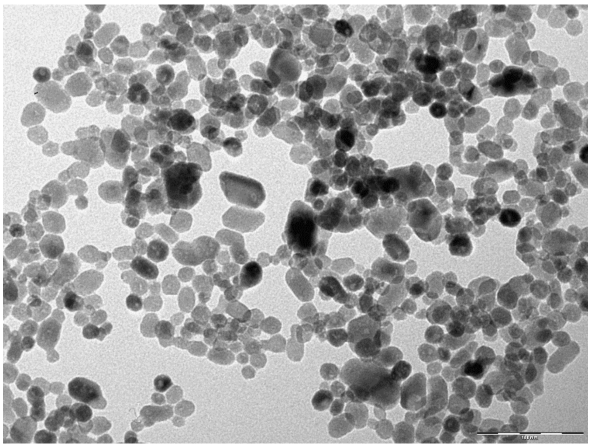

Fig. S8. Transmission electron microscopy (TEM) image of $CoFe_2O_4$/19 nm particles.

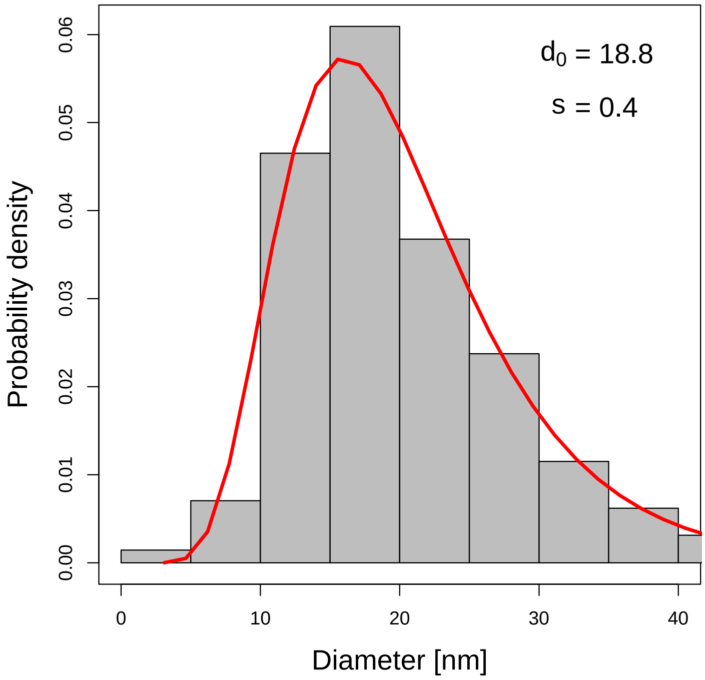

Fig. S9. Size distribution of $CoFe_2O_4$/19 nm particles. The red curve is a fit of a lognormal curve to the data.

Summary of the particle properties

A summary of the parameters of the different nanoparticles are listed in Table S1.

Table S1.

Nanoparticle reference	**Fe_2O_3/20 nm**	**$CoFe_2O_4$/45 nm**	**$CoFe_2O_4$/10 nm**	**$CoFe_2O_4$/19 nm**
Composition	γ-Fe_2O_3	$CoFe_2O_4$	$CoFe_2O_4$	$CoFe_2O_4$
Nominal core size [nm]	20	45	10	19
Lognormal size parameter d_0 [nm]	18.1	-	10.2	18.8
Lognormal width parameter σ [-]	0.43	-	0.39	0.4
Magnetic compound concentration (iron/cobalt) [mg/ml]	14.8	11.19	187.4	27.34
Initial concentration of the whole molecule [mg/ml]	21.16	14.66	262.55	31.66
Initial molar concentration of the whole molecule [mol/l]	0.13	0.062	1.12	0.14
Saturation magnetization at the initial concentration [A/m]	1630	1012	20780	2790
Saturation magnetization normalized to reference conc. 10mg/ml [A/m]	770	690	791	881

References

[1] Granqvist, C.G.; Buhrman, R.A. Ultrafine metal particles. *J. Appl. Phys.* **47**, 2200 (1976).

[2] Hugounenq, P.; Levy, M.; Alloyeau, D.; Lartigue, L.; Dubois, E.; Cabuil, V.; Ricolleau, C.; Roux, S.; Wilhelm, C.; Gazeau, F.; Bazzi,R. Iron oxide monocrystalline nanoflowers for highly efficient magnetic hyperthermia. *J. Phys. Chem. C* **116**, 15702 (2012).

[3] Spizzo, F.; Sgarbossa, P.; Sieni, E.; Semenzato, A.; Dughiero, F.; Forzan, M.; Bertani, R.; Del Bianco, L. Synthesis of ferrofluids made of iron oxide nanoflowers: Interplay between carrier fluid and magnetic properties. *Nanomaterials* **7**, 373 (2017).
